# Mechanism of GBE Combined with TP on the Effect of AMPK/SREBP-1C/ACC Pathway on Lipid Metabolism in Heat-Stressed Broiler Liver

**DOI:** 10.3390/vetsci12050424

**Published:** 2025-04-29

**Authors:** Chenyang Zhou, Haoxiang Chen, Xingyue Wu, Huili Dong, Siliang Feng, Yajin Tie, Zhanqin Zhao, Lifang Si

**Affiliations:** College of Animal Science and Technology, Henan University of Science and Technology, Luoyang 471000, China

**Keywords:** thermal stress, ginkgo biloba extract, tea polyphenols, AMP-activated protein kinase, hepatic lipid metabolism

## Abstract

The liver accounts for almost 95% of lipid metabolism in broilers and serves as a crucial metabolic organ. Stress, which occurs when broilers are exposed to a heated environment, inhibits liver metabolism, significantly impacting their growth. This experiment investigated the combination of GBE with TP to improve hepatic lipid metabolism in heat-stressed broiler chickens by inhibiting the AMPK/SREBP-1C/ACC pathway. Three hundred broilers were reared usually until 21 days and randomly divided into six groups, namely CON group, HS group, TP group (300 mg/kg), GBE100 group (GBE100 mg/kg + TP300 mg/kg), GBE300 group (GBE 300 mg/kg + TP 300 mg/kg), GBE600 (600 mg/kg + TP 300 mg/kg) groups, where the CON group was kept at 23 °C, and the HS group and the TP, GBE100, GBE300, and GBE600 groups of each medication group were kept at 35 ± 2 °C for 10 h per day. Liver and serum samples were extracted at 28 and 42 days of age, respectively. The results showed that, at 42 days of age, the GBE600 group exhibited significantly superior performance to the HS group in ADG, ADFI, and F/G (*p* < 0.01). Serum TG, TC, and LDL-C levels were significantly lower (*p* < 0.01), while HDL-C levels were significantly higher (*p* < 0.05). Additionally, the mRNA expression levels of LKB1, AMPK, SREBP-1C, and ACC were markedly reduced (*p* < 0.01). In contrast, the mRNA expression of HSL and CPT1A was significantly elevated (*p* < 0.01), indicating that the GBE600 was more effective in mitigating heat stress in broiler chickens at 42 days of age. It showed that the GBE600 was more effective in ameliorating heat stress in broilers at 42 days of age, thus providing an ethical basis for ameliorating the flocculation of hepatic lipid metabolism in heat-stressed broilers.

## Simple Summary

Prolonged heat stress can damage the hepatic lipid metabolism system of broiler chickens, leading to increased hepatic lipid deposition. This ultimately leads to metabolic flocculation, decreased growth performance, and an effect on broiler chickens’ growth and development. Therefore, it is of great significance to investigate the inhibitory effect of HS on the abnormalities of hepatic lipid metabolism and to reduce the damage caused by HS. In this experiment, Ginkgo biloba extract (GBE) was combined with tea polyphenols, and the optimal dosage of GBE in the combination was investigated to improve the HS induced imbalance of hepatic lipid metabolism and to promote the healthy growth of broilers.

## 1. Introduction

Heat stress (HS) is a significant factor impacting broiler performance. Despite the modernization of broiler farming to enhance the growth environment, broilers exhibit inadequate heat dissipation capabilities under high-temperature conditions, rendering them susceptible to heat stress. This phenomenon frequently occurs in tropical and subtropical regions, resulting in substantial economic losses for the poultry industry [1].

Genetic Screening Modern broilers are fast-growing, metabolically active, and produce high heat. Also have physiological characteristics such as abundant feathers and no sweat glands, which trigger the phenomenon of heat stress when the temperature exceeds 30 °C [2]. In elevated temperatures, broilers’ thermoregulatory centres are compromised, prompting them to combat heat stress by extending their necks and opening their mouths for respiration, consuming substantial quantities of water, and minimizing aggregation and feather loss. The liver is susceptible to heat stress, which results in increased blood flow to the liver region. This causes accelerated heat dissipation from the body surface, thus lowering the body temperature of broilers [3]. Simultaneously, the liver, being one of the most significant metabolic organs in the broiler’s body, is responsible for over 90% of lipid synthesis, encompassing fatty acid (FA) synthesis, triglyceride (TG) synthesis, the secretion and degradation of lipoproteins (LP), and the re-synthesis of triglycerides (TG), among other processes [4]. In high-temperature environments, broiler lipoprotein lipase activity diminishes, damaging liver cells. This damage disrupts lipid metabolism in the liver and serum, leading to fat accumulation and elevated cholesterol levels. Excess fat deposition in broilers and a cascade of metabolic disorders ultimately result [5]. Under heat stress, significant reactive oxygen species (ROS) are produced from lipid peroxidation in the hepatocytes of broiler chickens. This results in oxidative stress and overproduction of inflammatory factors, which causes hepatocellular injury, inflammation, apoptosis of hepatocytes, and, ultimately, the onset of NAFLD [6]. Under heat stress circumstances, broilers’ serum indices exhibit alterations characterized by increased levels of triglycerides (TG), high-density lipoprotein cholesterol (HDL-C), and low-density lipoprotein cholesterol (LDL-C) [7]. Heat stress diminishes the levels of nutrients, including vitamins and minerals, in the liver and serum of broiler chickens while also expelling minerals from the body, resulting in metabolic problems in the liver [8]. Extended heat stress in broilers can result in diminished growth rates, reduced meat yield, and elevated mortality rates, leading to significant economic losses for the agricultural sector [9].

Tea polyphenols (TP) are composed of polyphenolic compounds [10]. Catechins have the highest content, accounting for 60–80% of the total phenolic content. [11]. Catechin is the most biologically active component of tea polyphenols due to its unique chemical structure and wide range of biological activities [12]. JIN et al. demonstrated that the administration of tea polyphenols diminished subcutaneous fat accumulation in broiler abdominal chickens, decreased serum TG and total cholesterol (TC) concentrations, down-regulated genes associated with lipid anabolism, and up-regulated the expression of genes pertinent to hepatic fat transport and catabolism in liver tissues [13].

Ginkgo biloba extract (GBE) contains flavonoids, terpenoids, phenols, and other active constituents. Flavonoids are the most abundant, accounting for about 24% [14]. Ginkgo flavonoids primarily consist of chromogenic tungsten and chromogenic alkane derivatives, such as biflavonoid, bilobalamin, quercetin, and catechin. These flavonoids exhibit beneficial biological actions, including free radical scavenging, antioxidant properties, and antiviral effects [15]. Ginkgo biloba extract has anti-inflammatory, anti-allergic, hypolipidemic, memory enhancement, improvement of cardiovascular circulation, and liver protection effects on the body [16]. Adding 0.1% to 0.6% ginkgo biloba extract to heat-stressed broiler feed effectively protects the heart, liver, and intestinal tract from damage from heat stress [17]. Heat shock protein (HSP) is a crucial element for cellular defence. Under normal circumstances, HSP is expressed at low cell levels and increases upon thermal stimulation. HSP 70 is the most responsive to thermal stimulation, and its expression level directly indicates the extent of hepatic tolerance to elevated temperatures. Research indicates the activation of the AMPK pathway under hyperthermic conditions [18]. ACC, p-ACC, a substrate of AMPK, is frequently used to indicate AMPK activation in cells and tissues. AMPK phosphorylates ACC1/2 (Ser79/212) on serine residues, suppressing ACC activity and fatty acid synthesis. AMPK also directly phosphorylates SREBP-1c, which inhibits its cleavage and translocation. Furthermore, AMPK can directly phosphorylate SREBP-1c, preventing its nuclear translocation and thereby decreasing SREBP-1c expression and lipid synthesis [19,20]. AMPK inhibits lipid breakdown by phosphorylating Ser565 at the HSL site [21]. The AMPK/SREBP-1c/ACC pathway is crucial for lipid synthesis regulation; its activation can mitigate insulin resistance-induced lipid peroxidation and buildup in liver tissues, diminishing liver harm [22].

The extensive usage of antibiotics in recent years has rendered their residues increasingly detrimental to animal organisms. Traditional Chinese medicine, characterized by its absence of residue, non-toxicity, and lack of resistance, is increasingly being integrated into the livestock business [23]. The present dietary supplementation of 300 mg/kg tea polyphenols (TP) led to a consistent decrease in egg yolk cholesterol [24,25]. The comedication group was modelled by using a gradient dose (100, 300, and 600 mg/kg) of GBE with a fixed dose (300 mg/kg) of TP that was added uniformly in equal proportions to the basal feed. Growth performance, liver pathology assessment, pertinent serum indices, and quantitative PCR were conducted on broilers aged 28 and 42 days. To explore the optimal concentration of GBE added to improve the hepatic lipid metabolism of broiler chickens under heat stress in the combination of drugs, to provide theoretical references for broiler chickens in improving hepatic lipid metabolism under heat stress, to improve the ability of broiler chickens to metabolize nutrients.

## 2. Materials and Methods

### 2.1. Experimental Design

The experimental animals were purchased from 1-day-old white-feathered broilers from Jinhua Meat Breeding Farm, Tangyin County, Anyang City, China and reared them conventionally until they reached 21 days of age. Three hundred broilers of comparable body weights were selected and randomly allocated into six groups, each including five replicates, with ten birds per replicate. CON group; HS group; TP group (TP 300 mg/kg); GBE100 group (GBE 100 mg/kg + TP 300 mg/kg); GBE 300 group (GBE 300 mg/kg + TP 300 mg/kg); GBE 600 group (GBE 600 mg/kg + TP 300 mg/kg). The CON ground was maintained at 23 ± 2 °C, whereas the HS group and each medication group were maintained at 35 ± 2 °C. The humidity in the chicken house ranged from 55% to 60%, and heat stress was administered for 10 h daily, from 9 AM to 7 PM. The CON and HS groups were fed a basal diet. The TP group was fed a basal diet supplemented with 300 mg/kg of TP, the GBE100 group was fed a basal diet supplemented with (GBE 100 mg/kg + TP 300 mg/kg), the GBE300 group was fed a basal diet supplemented with (GBE 300 mg/kg + TP 300 mg/kg), and the GBE600 group was fed a basal diet supplemented with (GBE 600 mg/kg + TP 300 mg/kg). All broilers were assured a consistent feed and water supply during the experiment. GBE was procured from Osaka Pharmaceutical Co. Ltd., Osaka, Japan, while TP was acquired from YINO Biotechnology Co., Hangzhou. China. The experimental animals received approval from the Animal Ethics Committee of Henan University of Science and Technology, Luoyang, China.

### 2.2. Basic Rations

Acquired from Vanda broiler litter feed in Meng Jin County, Luoyang City, the composition and nutrient content of the foundational ration are presented in Table 1.

### 2.3. Sample Collection

Sampling occurred at 28 and 42 days of age, respectively, with broilers fasting for 12 h prior, and net body weight was recorded. Blood was extracted from the vein beneath the chicken wing, centrifuged at 3500 rpm for 5 min, and the serum was subsequently stored in a refrigerator at −40 °C. Chickens were euthanized via intravenous injection of sodium barbiturate following blood collection, and liver samples were promptly obtained. The samples were preserved in two sections, with the initial sample immersed in 4% paraformaldehyde. The second half was frozen in liquid ammonia and stored at −80 °C.

### 2.4. Measurement Methods

#### 2.4.1. Measurement of Growth Performance

Broilers were raised conventionally until 21 days of age, at which point they were weighed for the first time and then assigned to random groups. Daily measurements of body weight and feed consumption for each group of broilers were conducted punctually. Daily feed intake, mean daily weight growth, and feed conversion ratio were measured after a 12-h fasting period before weighing, utilizing the subsequent equations.

Average Daily Food Intake (ADFI) = (Feedstock—Remnant)/Experimental Days

Average Daily Gain (ADG) = (Final Phase—Primordial)/Experimental Days

Feed Conversion Ratio (FCR) = Average Daily Food Intake/Average Daily Gain

#### 2.4.2. Histopathological Changes in the Liver

Liver tissue was extracted from 4% paraformaldehyde, sectioned into small fragments (about 6 mm × 6 mm × 4 mm), placed in an embedding frame, and rinsed under running water for 8 h. Following a gradient concentration protocol, it was submitted to ethanol dehydration, xylene clearing, and paraffin embedding. The implanted blocks were positioned in a slicer and sectioned to a 4–6 µm thickness, mounted on slides in a water bath at 42–45 °C, dried at 37 °C, and stained following the protocol for hematoxylin and eosin. The liver’s histopathological alterations were examined under a light microscope.

#### 2.4.3. Hepatic Lipid Area Analysis

Liver tissue was extracted from 4% paraformaldehyde, washed with PBS, cryo-embedded using a freezing embedding agent (OTC-Japan-Sakura), frozen at −20 °C until fully set, stored in a freezer (Leica CM1950), and sectioned to a thickness of 6–8 µm for slide application. Oil red staining was conducted following the specified protocol, revealing reddish staining of lipid deposition under a light microscope, with the degree of lipid accumulation assessed using ImageJ 1.53t.

#### 2.4.4. Measurement of Serum Lipid Content

The serum of broilers at 28 and 42 days of age was extracted individually, and TG, TC, HDL-C, and LDL-C were quantified following the kit instructions from the Nanjing Jian Cheng Institute of Biological Engineering (Nanjing, China). The kits were acquired from the Nanjing Jian Cheng Bioengineering Institute (Nanjing, China).

#### 2.4.5. Expression of Genes Related to Hepatic Lipid Metabolism

Total RNA was isolated from the livers of 28 and 42-day-old broilers using the TRIzol method. The cDNA was reverse transcribed and diluted fivefold to prepare samples, with three replicate groups established for each sample. Gene expression was quantified using fluorescence quantitative PCR, and relative quantification of the target gene was conducted by the 2−ΔΔCt method, with β-actin as the internal reference gene. RNA extraction kit, reverse transcription kit, and Cham Q Universal SYBR qPCR Master Mix were acquired from Novozymes Bioscience Co. (Tianjin, China). Primer sequences are presented in Table 2.

### 2.5. Statistics and Analysis of Data

Data were examined utilizing one-way ANOVA and T-test with IBM SPSS Statistics 26 software, while GraphPad Prism 9.00 program was employed for graphical representation. A *p*-value of less than 0.05 indicated a statistically significant difference.

## 3. Results

### 3.1. Production Performance Measurement

A comparison of ADG, ADFI, and feed F/G in Table 3 indicates that the. The disparity was markedly significant in the HS group relative to the CON group (*p* < 0.01); The TP group alone and the GBE100 group in combination, respectively, showed significant differences compared with the HS group (*p* < 0.05). The disparity was more pronounced (0.01 < *p* < 0.05). The difference was even more pronounced in the combined GBE600 group compared to the HS group (*p* < 0.01).

### 3.2. HE Pathologic Observation of Liver Tissue

**Figure 1:**
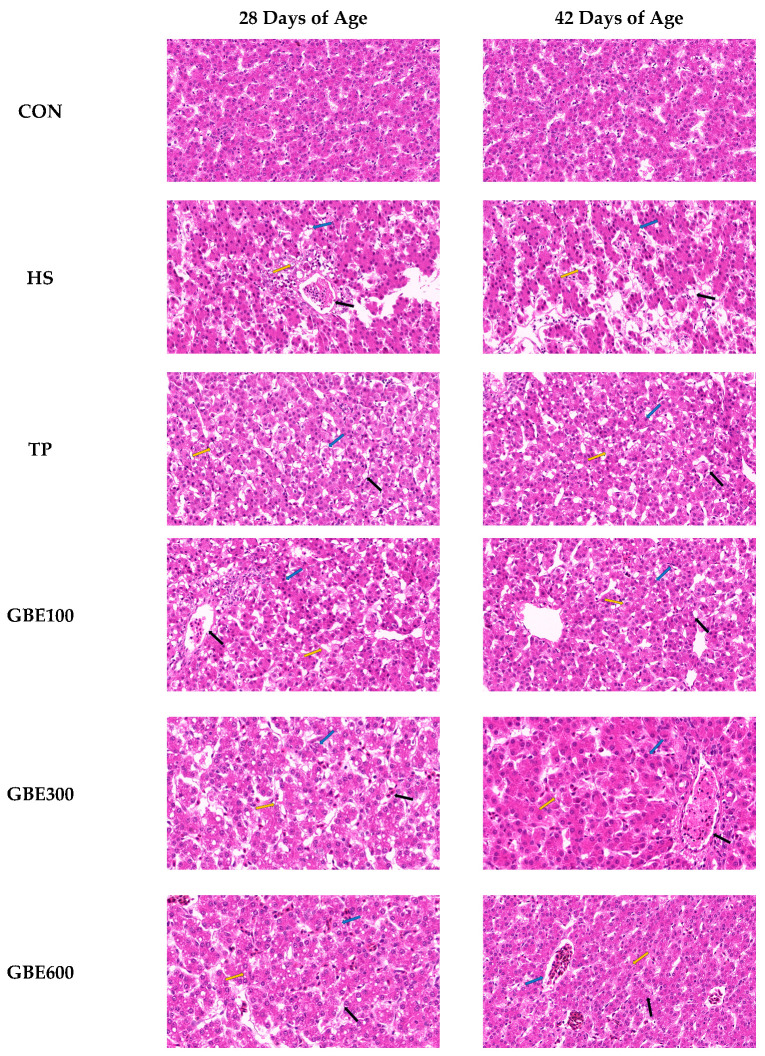
Broiler liver HE pathology section observation (magnification 400), Blue arrow: hepatocytes, yellow arrow: represents lipid droplets, black arrow: hepatic blood sinusoids.

Figure 1 illustrates that, at 28 days of age, the HS group had clustered lipid droplets inside the cytoplasm of hepatocytes, marginalized nuclei, pronounced cytoplasmic hyalinosis, and a significant increase in hepatic sinusoids compared to the CON group, compared to the HS group, the TP and GBE100 groups exhibited no significant alterations in hepatocyte morphology. In contrast, the GBE300 and GBE600 groups demonstrated a modest reduction in lipid droplets within hepatocytes, a centralization of the nucleus, and a partial restoration of cytoplasmic hyalinosis relative to the HS group. At 42 days of age, the HS group had augmented honeycomb-like densities in the hepatocytes, with the nuclei significantly extruded on one side in contrast to the CON group. The TP group and the GBE (100 and 300) group exhibited a gradual reduction in lipid droplet accumulation, a decrease in hepatocyte swelling, a tendency for nuclear centering, and a reduction in the hyaline phenomenon. In contrast, the GBE600 group demonstrated a significant reduction in hepatocyte lipid droplets, with the nucleus centrally positioned, a marked disappearance of the honeycomb phenomenon, and increased cytoplasmic concentration.

### 3.3. Liver ORO Lipid Observations

**Figure 2:**
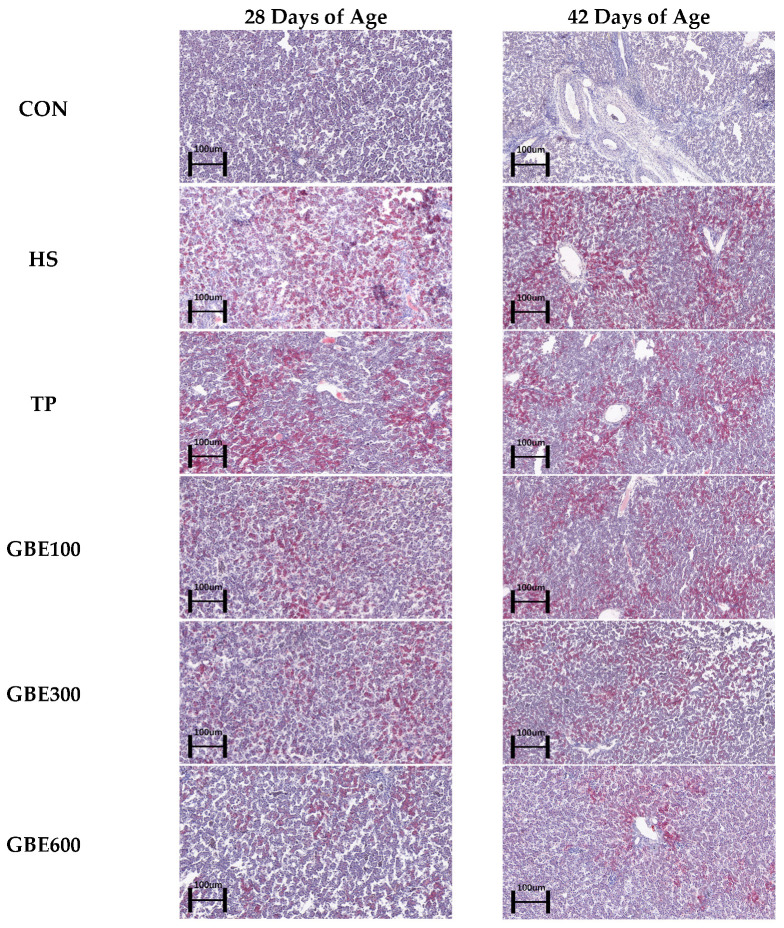
ORO observation of liver fat deposition in broiler chickens (magnification 100).

**Figure 3:**
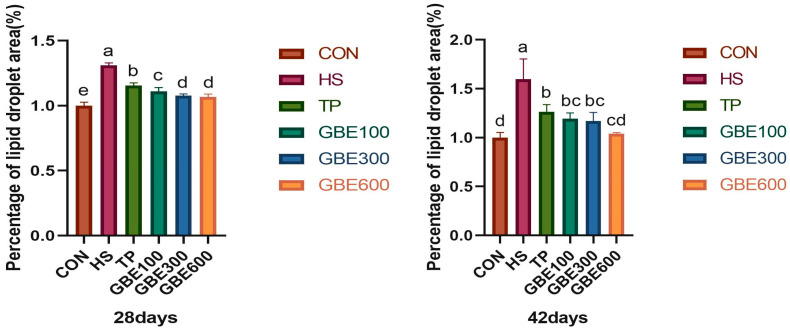
Liver lipid droplet area analysis by ImageJ. Note: Peer shoulder markings with identical letters denote non-significant differences (*p* > 0.05). Adjacent letters signify significant differences (*p* < 0.05), whereas letters separated by one indicate very significant differences (*p* < 0.01).

Figure 2 illustrates the region of lipid accumulation in the heat-stressed liver result-ing from the combination of GBE and TP. Through ImageJ study of oil red staining, Figure 3 illustrates a significant difference in hepatic fatty deposits between the HS and the CON groups at 28 compared to 42 days of age (*p* < 0.01). The area of hepatic lipid deposition was significantly diminished in the TP group relative to the HS group (*p* < 0.05). Furthermore, the area of hepatic fat deposition was markedly reduced in the combined drug GBE100 group compared to the TP group (*p* < 0.05). Additionally, as the dosage of the combined drug increased in the GBE (300, 600) group, the area of hepatic fat deposition experienced a highly significant reduction (*p* < 0.01). At 42 days of age, the area of hepatic lipid deposition was significantly less in the TP group and the combined drug (100, 300) group compared to the HS group (*p* < 0.05), while the difference in the GBE600 group compared to the HS group was highly significant (*p* < 0.01).

### 3.4. Serum Lipid Profile Test

**Figure 4:**
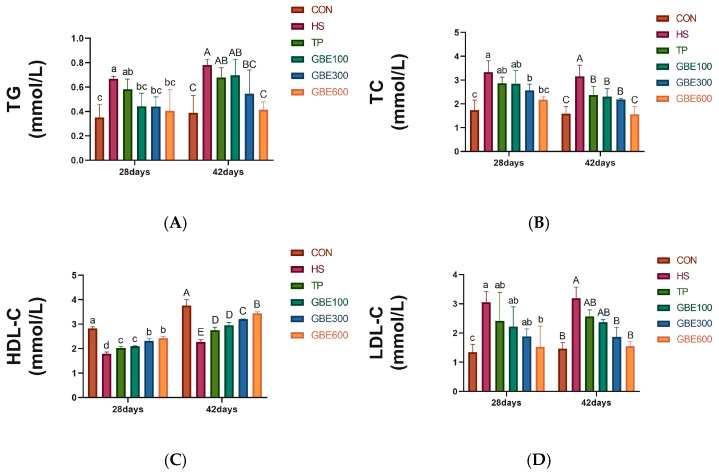
(**A**) graph of TG changes in serum. (**B**) graph of TC changes in serum. (**C**) graph of HDL-C changes in serum. (**D**) graph of LDL-C changes in serum. Note: Peer shoulder markings with identical letters denote non-significant differences (*p* > 0.05). Adjacent letters signify significant differences (*p* < 0.05), whereas letters separated by one indicate very significant differences (*p* < 0.01).

Figure 4 illustrates that the disparities in TG, TC, LDL-C, and HDL-C between the HS group and the CON group at 28 and 42 days of age were statistically significant (*p* < 0.01); At 28 days of age, the differences in TC, TG, and LDL-C were not signif-icant (*p* > 0.05), HDL-C was significant (*p* < 0.05) in the TP alone group compared to the HS group; The disparity in TC and LDL-C was not significant (*p* > 0.05), however the difference in TG and HDL-C was substantial (*p* < 0.05) in the combined medica-tion GBE100 group compared to the HS group; The differences in TG, TC, and HDL-C were substantial (*p* < 0.05), however the difference in LDL-C was not significant (*p* > 0.05) in the co-administered GBE300 group compared to the HS group; The difference in TG, TC, HDL-C, and LDL-C between the combined GBE600 phase and HS group was significant (*p* < 0.05); At 42 days of age, the differences in TG and LDL-C were not significant (*p* > 0.05), whereas HDL-C and TC were significant (*p* < 0.05) in the TP group alone and the GBE100 group in conjunction with the HS group. The difference in TG, TC, HDL-C, and LDL-C was significant in the co-administered GBE300 group compared with the HS group (*p* < 0.05). The differences in TG, TC, and HDL-C were highly significant (*p* < 0.01), whereas LDL-C was substantial (*p* < 0.05) in the combined medication GBE600 group compared to the HS group.

### 3.5. Determination of mRNA for Lipid Metabolism-Related Genes

**Figure 5:**
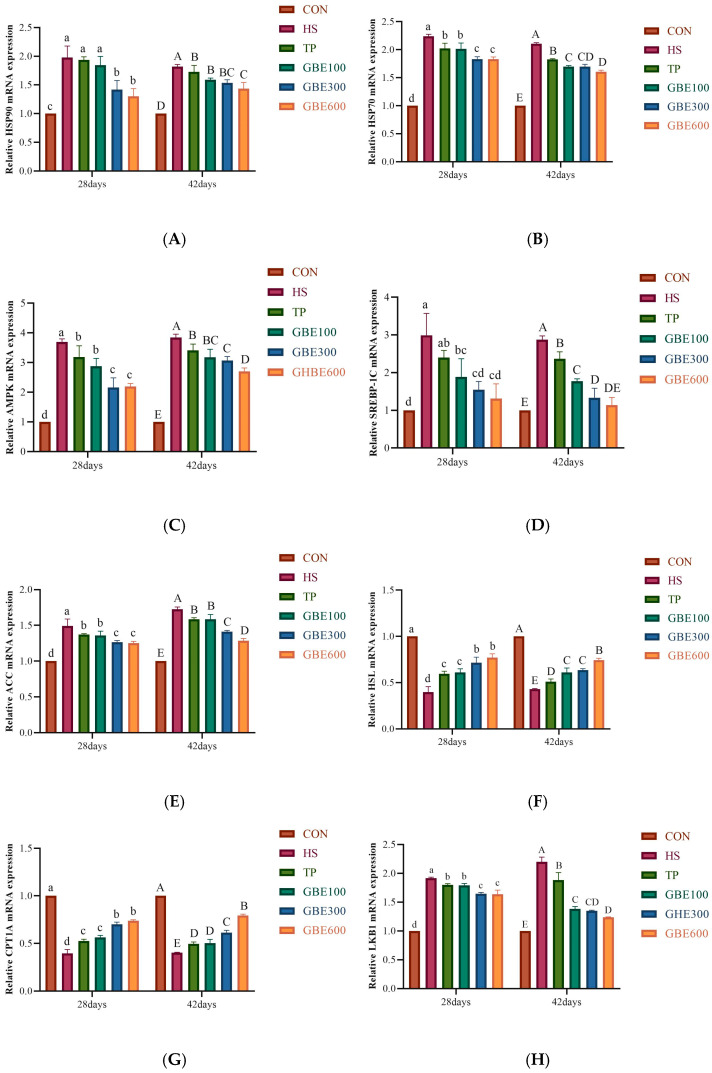
(**A**) Hepatic HSP 90 mRNA trend. (**B**) Hepatic HSP 70 mRNA trend. (**C**) Hepatic AMPK mRNA trend. (**D**) Hepatic SREBP-1C mRNA trend. (**E**) Hepatic ACC mRNA trend. (**F**) Hepatic HSL mRNA trend. (**G**) Hepatic CPT1A mRNA trend. (**H**) graph: trend of hepatic LKB1 mRNA change. Note: Peer shoulder markings with identical letters denote non-significant differences (*p* > 0.05). Adjacent letters signify significant differences (*p* < 0.05), whereas letters separated by one indicate very significant differences (*p* < 0.01).

Relative expression of HSP90 (A), HSP70 (B), AMPK (C), SREBP-1C (D), ACC (E), HSL (F), CPT1A (G), and LKB1 (H) in each dosing group before and after heat stress and under heat stress was detected by q-PCR. The results, as shown in Figure 5, showed that the relative expression of HSP90 (A), HSP70 (B), AMPK (C), SREBP-1C (D), ACC (E), and LKB1 (H) was elevated in the HS group compared with the CON group at 28 versus 42 days of age. The difference was highly significant (*p* < 0.01); the relative expression levels of HSL (F) and CPT1A (G) were significantly lower than those of the CON group, with highly significant differences (*p* < 0.01). The relative expression of HSP90 (A) at 28 days of age indicated that the difference between the TP group alone and the combined GBE100 group compared to the HS group was not significant (*p* > 0.05); however, the difference between the combined GBE300 and GBE600 groups compared to the HS group was significant (*p* < 0.05). The relative expression of HSP90(A) at 42 days of age was considerably different in the TP group and the combination (GBE100, GBE300) group compared to the HS group (*p* < 0.05), with a highly significant difference observed in the combined GBE600 group relative to the HS group (*p* < 0.01). At 28 days of age, the ex-pression levels of HSP70 (B), AMPK (C), ACC (E), CPT1A (G), and LKB1 (H) exhibited significant differences between the TP group alone and the GBE100 group in conjunction with the HS group (*p* < 0.05), the differences between the combined groups (GBE300 and GBE600) and the HS group were highly significant (*p* < 0.01); At 42 days of age, the disparity between the TP group alone and the combined GBE100 group versus the HS group was significant (*p* < 0.05). The difference between the combined GBE300 group and the HS group was even more significant (0.01 < *p* < 0.05), as was the comparison of the combined GBE600 group with the HS group. The difference was statistically significant compared to the HS group (*p* < 0.01). The relative expression of HSL(F) at 28 days of age exhibited a significant difference (*p* < 0.05) in the TP group and the GBE100 group in combination when compared to the HS group, whereas the difference in the combination of the GBE300 and GBE600 groups was very significant (*p* < 0.01) relative to the HS group; At 42 days of age, the disparity between the TP and GBE100 groups and the HS group was significant (*p* < 0.05), while the gap between the GBE300 group and the HS group was even more pronounced (0.01 < *p* < 0.05); The difference was highly significant (*p* < 0.01) in the combined GBE600 compared to the HS group.

## 4. Discussion

Chronic heat stress adversely affects poultry growth performance, leading to elevated feed intake, diminished body weight, and an increased feed-to-weight ratio [26,27]. The experimental results indicated that heat stress adversely affected broiler growth, reducing feed intake and body weight. At the same time, the feed-to-weight ratio increased, signifying that heat stress detrimentally impacts broiler growth and feed-to-meat conversion efficiency by prior research. As the content of Ginkgo biloba extract increased, the F/G significantly decreased, and the ADG and ADFI significantly increased. This experiment demonstrates that, after prolonged medication, the efficacy of the GBE100 and TP groups is comparable at 42 days of age. The combined groups with higher GBE dosages (GBE300 and GBE600) exhibit superior effects compared to the separate TP group, with the GBE600 group showing the most pronounced efficacy. This indicates that for broilers, enhancing resistance to heat stress, increasing feed intake, and improving the meat-to-feed ratio is highly beneficial. The most significant effect was observed in the GBE600 group.

Heat stress in poultry disrupts hepatic lipid metabolism, impairing the liver’s capacity to process free fatty acids, leading to lipid accumulation and ultimately overburdening liver function [28]. The liver is a crucial organ for lipid absorption and catabolism in broiler chickens; yet, during heat stress, the body cannot effectively mobilize fat for energy, leading to hepatic lipid metabolism flocculation [29]. Fat accumulation occurred in chicken liver due to heat stress, which was stained into large red areas by oil red O staining [30]. In this experiment, the area of lipid droplets stained by hepatic ORO oil red staining was significantly elevated in the HS group at 28 days of age, indicating a substantial impairment in broilers’ hepatic lipid metabolism capacity under heat stress. Furthermore, the area of hepatic lipid accumulation increased with age, suggesting that heat stress exacerbated liver damage, resulting in excessive lipid accumulation in the liver and adversely affecting hepatic lipid metabolism capacity. This is consistent with previous studies. The combined drug group (GBE100, GBE300, GBE600) exhibited superior results compared to the separate drug group (TP group). At 28 days of age, the combination drug group (GBE300, GBE600) showed equivalent efficacy in mitigating heat stress. It may be related to the fact that broiler liver is more damaged than the therapeutic effect of drugs under heat stress, or it may be related to the health of the body’s intestinal tract, which is subjected to a large number of endogenous and exogenous stimuli during heat stress, which hinders the digestion and absorption of nutrients and drugs [31]. At 42 days of age, the prolonged medication administration reveals that the combined medication groups (GBE100, GBE300) exhibit similar effects on broiler resistance to thermal environmental stress, while the GBE600 group demonstrates the most significant efficacy. This suggests that extended use of the combined medication, particularly GBE600, markedly enhances lipid metabolism regulation in the liver, thereby supporting optimal hepatic function.

Blood biochemical indicators are an important means of detecting animal health status and can also indirectly reflect the degree of adaptation of animals to the ecological environment [32]. Long-term heat stress on the animal body is often accompanied by lipid metabolism flocculation, causing dyslipidemia. This is mainly characterized by an increase in TC, TG, and LDL-C content and a decrease in HDL-C concentration. Long-term abnormal lipid metabolism damages the liver and leads to metabolic diseases. [33]. TC and TG are components of blood lipids, and their levels reflect lipid metabolism [34]. HDL-C and LDL-C are important markers for detecting hepatic prognostic therapy and liver function, respectively [35]. In this experiment’s HS group of broiler chickens, serum TG, TC, and LDL-C levels were elevated. HDL-C was decreased, consistent with the manifestation of dyslipidemia, which was in agreement with previous studies. Following administration, the efficacy of the GBE combined group surpassed that of the TP group alone. With prolonged dosing duration, at 42 days of age, the GBE600 group demonstrated significant effects on regulating TG, TC, HDL-C, and LDL-C in serum, ameliorated serum lipid abnormalities, and enhanced hepatic lipid metabolism efficiency. Ginkgolide B is a key component of Ginkgo biloba with unique physiological activity, which mainly activates blood circulation, removes blood stasis, reduces fat, and eliminates stagnation [36,37]. Ginkgo biloba extract at a 0.24% additive dose significantly reduces liver fat in broilers [38]. It was shown that high concentrations of ginkgo biloba and tea polyphenols for coadministration favorably affected hepatic lipid metabolism clearance in heat-stressed broilers.

Inducible HSP70 is one of the most important proteins of the heat shock family; it appears only in stressed cells, and the level of HSP70 mRNA transcription is higher than that of the standard group during heat stress [39]. The relative expression of HSP70 and HSP90 indicated the liver’s stress resistance, and under heat stress, their expression levels were elevated compared to the standard group [40]. The HSP70 and HSP90 mRNA expression levels were elevated in the HS group in this experiment, aligning with other research findings. Heat stress leads to lipid peroxidative stress in the liver, causing a dynamic imbalance in hepatic lipid metabolism. Ginkgo biloba extract has good antioxidant activity, inhibits free radicals and lipid peroxidation, and reduces heat stress damage to the body [41]. In the GBE comedication cohort, a notable reduction in HSP70 and HSP90 mRNA levels was observed in the GBE300 and GBE600 groups at 28 days of age compared to the HS group. Furthermore, with extended medication duration, a significant decline in HSP70 and HSP90 mRNA was evident in the GBE600 group at 42 days of age, indicating that prolonged treatment in the GBE600 group enhanced hepatocyte resistance to heat stress and improved stability.

AMPK is responsible for regulating body energy and is the cell’s primary energy receptor and metabolic sensor; long-term heat stress significantly alters hepatic fat metabolism in broilers [42]. Heat stress induces aberrant energy metabolism within the body, wherein LKB1 activates AMPK through the phosphorylation of its α-subunit. Most of AMPK’s regulation of fatty acid oxidation metabolism occurs via the phosphorylation and inhibition of ACC activity. This results in decreased malonyl-coenzyme A levels and enhanced expression of genes associated with fatty acid oxidation [43]. Carnitine palmitoyl transferase (CPT) is critical in mitochondrial β-oxidation and is responsible for free fatty acid metabolism. CPT1A is specifically expressed in the liver, and upregulation of CPT1A improves NAFLD in mice [44,45]. AMPK is activated, and downstream ACC phosphorylation leads to ACC inactivation, favoring CPT1A fatty acid mitochondrial β-oxidation [46]. Heat stress leads to a rise in SREBP-1C expression and increased fat synthesis in broilers, and this effect can be attenuated by inhibiting SREBP-1C to maintain lipid metabolism homeostasis [42]. HSL is a crucial enzyme in lipolysis, facilitating the liberation of hydrolyzed fatty acids from triglycerides and diacylglycerides in adipose tissue. Furthermore, it has been demonstrated that heat stress diminishes mRNA expression of genes in pig subcutaneous adipose tissue [47]. In this experiment, in the HS group at 28 days of age, the mRNA expression contents of LKB1 and AMPK were abnormally elevated, which indicated that the liver was subjected to oxidative damage caused by heat stress, resulting in the elevation of the mRNA contents of SREBP-1C and ACC, which strengthened the ability of TG and TC synthesis in the liver, which was verified with the related indexes detected in serum in the present experiment. HSL and CPT1A are important indicators of lipid oxidative catabolism, and the decrease in HSL and CPT1A mRNA expression in the 28-day-old HS group in this experiment indicates that the inhibition of hepatic lipolytic capacity will result in the elevation of hepatic lipid content. At 42 days of age. The HSL and CPT1A mRNA contents in the HS group were still in decline compared with the CON group, although there was an inevitable increase in the HSL and CPT1A mRNA contents. It indicates that the organism has a restorative effect on hepatic lipid oxidation, but the effect is minimal. In each dosing group, at one week of dosing (28 days of age), it was evident from the mRNA expression of LKB1, AMPK, SREBP-1C, ACC, HSL, and CPT1A that the GBE300 group exerted the same efficacy as the GBE600 group. It may be related to the fact that Chinese medicines usually have multitarget effects, and GBE300 and GEB600 may regulate the AMPK pathway at the same time, thus producing similar effects in the short term. It may also be related to the fact that heat stress is an acute stress-damaging mechanism, and under heat stress, the broiler organism may be susceptible to the intervention of traditional Chinese medicines, reflecting consistently the intervention of different concentrations of GBE300 and GEB600 in the short term, to make the organism reach the maximum regulatory capacity and produce similar short-term therapeutic effects. After long-term medication (42 days of age), the GEB600 group had a favorable regulation of hepatic lipid metabolism in heat-stressed broilers. This may be related to the fact that the active ingredients of the drugs accumulated in the broilers, which improved the microcirculation of the body and reduced the adverse effects of heat stress on it. It may also be related to the fact that Chinese veterinary drugs regulate the balance of qi and blood in the body, scavenge free radicals, and enhance the resistance of the liver to heat stress.

## 5. Conclusions

This study confirmed that GBE combined with TP (GBE100, GBE300, and GBE600 groups) significantly modulates hepatic lipid metabolism flocculation under broiler heat stress in the heat stress broiler model. The GBE600 group showed the best recovery effect at 42 days of age, which was reflected in the improved growth performance of broilers, significant reduction of hepatic lipid accumulation, and normalization of lipid metabolism indexes, accompanied by the inhibition of lipid synthesis genes AMPK, SREBP-1C, and ACC, and the activation of lipolysis genes HSL and CPT-1A. This suggests that the combination of high dose GBE and TP may be regulated through the AMPK/SREBP-1C/ACC signaling pathway, thus reversing the imbalance of lipid metabolism caused by heat stress and providing a theoretical basis for the nutritional regulation strategy of heat stress syndrome in broiler chickens in breeding. Although the present experiment focused on heat stress on hepatic lipid metabolism capacity in broilers, the effects of lipid metabolism capacity in other metabolic organs (intestines, adipose tissue) were not assessed, possibly underestimating the role of the GBE600 in the systemic regulation of the broiler organism.

## Figures and Tables

**Table 1:** Composition of Ration and Nutritional Content.

Raw Material	Content (%)
Soybean Meal	15–30
Maize	60–65
Stone Powder	1–5
Talc	0.8–1
Calcium Bicarbonate	0.5–1
Composite Enzyme	0.03–0.05
Vitamin Complex Premix	1.5–2
Amino Acids	1.5–2
Crude Protein ≥	21.0
Total Phosphorus ≥	0.5
Methionine ≥	0.3–0.8
Crude Fiber ≤	5.0
Crude Ash ≤	8.0
Moisture ≤	14.0
Edible Salt	0.3–0.8
Soybean Meal	15–30
Maize	60–65

**Table 2:** Primer Sequences.

Genetics	Sequences (5′-3′)	Number
β-actin F	CCGCTCTATGAAGGCTACGC	20
β-actin R	CTCTCGGCTGTGGTGGTGAA	20
AMPK F	ATCTGTCTCGCCCTCATCCT	20
AMPK R	CCACTTCGCTCTTCTTACACCTT	23
SREBP1-c F	GAGACCATCTACAGCTCCGC	20
SREBP1-c R	CATCCGAAAAGCACCCCTCT	20
ACC F	TCCAGCAGAACCGCATT	17
ACC R	GTATGAGCAGGCAGGACTT	19
LKB1 F	AGACTCTGGTGCCCATACCT	20
LKB1 R	CTCAGGCACCTGTCCTGGTA	20
HMGCR F	GCGGCAGATTTGCTGACTG	19
HMGCR R	TGGGCACTCATAGTTCCAGC	20
CPT1A F	GCATTGACCGCCATCTGTTC	20
CPT1A R	CAGGTCCAAATCCACCACCA	20
HSP70 F	ACAGTGCCCGCTTACTTCAA	20
HSP70 R	ACACATCAAAAGTGCCCCCT	20
HSP90 F	GGACCAACCAATGGAGGAGG	20

**Table 3:** Growth performance measurements.

	GROUPS					
	CON	HS	TP	GBE100	GBE300	GBE600
21 Weight (G/Only)	859.4 ± 20.12	869.6 ± 29.71	871 ± 25.3	865 ± 34.69	872.8 ± 23.44	887.2 ± 27.69
42 Weight (G/Only)	2556.4 ± 20.12 a	1936 ± 29.71 d	2187.4 ± 25.3 c	2210.2 ± 34.69 c	2421.8 ± 23.44 b	2508.4 ± 27.69 a
ADG (G/D)	80.81 ± 3.38 a	50.78 ± 3.02 c	62.69 ± 4.5 b	64.01 ± 4.26 b	68.24 ± 4.81 b	78.57 ± 2.47 a
ADFI (G/D)	86.66 ± 1.13 a	67.78 ± 1.5 e	72.16 ± 0.69 d	73.21 ± 1.42 d	76.71 ± 1.4 c	82.68 ± 0.95 ab
(F/G)	1.07 ± 0.14 d	1.33 ± 0.03 a	1.15 ± 0.11 b	1.14 ± 0.02 bc	1.12 ± 0.02 c	1.05 ± 0.12 d

Note: Peer shoulder markings with identical letters denote non-significant differences (*p* > 0.05). Adjacent letters signify significant differences (*p* < 0.05), whereas letters separated by one indicate very significant differences (*p* < 0.01).

## Data Availability

The data presented in this study are available on request from the corresponding author.
